# Triumphs of Mesmerism

**Published:** 1844-12-28

**Authors:** 


TRIUMPHS OF MESMERISM.

A medical gentleman of this place informs me, that he can at any time relieve spinal irritation, by mesmerism; also, that it is one of the best means of preventing abortion which he has ever tried. He can mesmerize a nervous patient after lying down for the night, and she will sleep soundly till he awakes her, which he can do by an effort of his will, transmitted from his own house. Still further, he can prevent the paroxysm of an intermittent fever, by mesmerizing his patient before the access of the chill, but the next paroxysm will come on. However, he has discovered (taking the hint from a work on homæopathy,) that by producing an artificial chill during the intermission a radical cure is effected. This he accomplishes by producing mesmeric sleep, and then applying his finger to the tip of the patient's nose. Finally, the following case has lately occurred in his practice. A lady was subject to such obstinate constipation, that she passed fourteen days without evacuation, when a drachm of calomel and six drops of croton oil produced only one discharge. Eight days after that, not having had another, he threw her into the mesmeric sleep, when mesmerizing a glass of water, he gave it to her as a solution of Epsom salts, the taste of which she admitted: he then made her hold half an ounce of calomel in her hand for about 30 minutes, when he awoke her. I do not recollect whether he mesmerized the calomel. When he called the next morning, he found that she had been purged to excess, and was profusely salivated, with swollen jaws and a mercurial breath. He then mesmerized her again, and putting a roll of brimstone (not mesmerized) into her hand, and applying another to her cheek, she was perfectly well in three days, and her costiveness has not since returned.

